# Phase-Dependent Transcriptional Reprogramming of *Vitis vinifera* During Pierce’s Disease Progression by *Xylella fastidiosa* Infection

**DOI:** 10.3390/ijms262211040

**Published:** 2025-11-14

**Authors:** Raghuraman Pandiyan, Seonjoo Park

**Affiliations:** Department of Life Sciences, Yeungnam University, Gyeongsan 38541, Gyeongsangbuk-do, Republic of Korea; praghuramanbiophysics@gmail.com

**Keywords:** *Vitis vinifera*, *Xylella fastidiosa*, Pierce’s disease, RNA-seq, differential gene expression, network analysis, hub genes, transcriptional reprogramming

## Abstract

Pierce’s disease (PD), caused by the xylem-limited bacterium *Xylella fastidiosa*, poses a significant threat to global grapevine (*Vitis vinifera*) production. Despite its economic importance, the dynamic molecular mechanisms underlying grapevine responses to infection remain poorly understood. This study re-analyzed the publicly available RNA-seq dataset GSE152164 to characterize phase-dependent transcriptional reprogramming during PD progression. Differential expression analysis using DESeq2 identified 1093 differentially expressed genes (DEGs) during the early infection phase (Phase I) and 136 in the intermediate phase (Phase II), indicating a strong early defense response followed by transcriptional downregulation as symptoms progressed. Comparative analysis distinguished 991 Phase-I-specific and 34 Phase-II-specific genes, along with 167 infection-specific temporal DEGs, underscoring a coordinated early immune response and subsequent metabolic repression. Protein–protein interaction network analysis identified 21 high-confidence hub genes, including chitinase (VIT_16s0050g02220), thaumatin-like protein (VIT_02s0025g04250), and EDS1 (VIT_17s0000g07560), which represent core regulators of defense and stress adaptation pathways. Collectively, this study elucidates the transcriptional dynamics underlying *V. vinifera* responses to *X. fastidiosa* and provides valuable insights for developing disease-resistant cultivars to mitigate Pierce’s disease.

## 1. Introduction

Plant–pathogen interactions play a critical role in determining plant health, crop productivity and ecosystem stability. A comprehensive understanding of these interactions is essential for developing effective disease control strategies and ensuring global food security [1].

*Xylella fastidosa* is a Gram-negative bacterium that colonizes the xylem, the tissue responsible for water transport in plants, and causes devasting diseases in several crops. In grapevines (*Vitis vinifera*), it is the etiological agent of Pierce’s disease (PD), characterized by leaf scorch, wilting, and progressive decline, often leading to plant death [2]. PD disrupts water transport and induces progressive physiological deterioration in *V. vinifera*. The pathosystem involves bacterial colonization of xylem vessels, biofilm formation, and vessel occlusion, collectively causing water stress, leaf scorch, and tissue necrosis [3]. Infection triggers the plant to form tyloses and other xylem blockages that interrupt water flow and impose severe physiological stress. PD is a major concern for grape production worldwide, such as in Majorca (Spain) and Taiwan and possibly spreading to the northern (Europe and China) and southern hemisphere (Chile, Argentina, South Africa, Australia, and New Zealand) [4], particularly in California, where it causes substantial economic losses estimated at over USD 100 million [5]. The pathogen is transmitted by xylem-feeding insects, and biofilm formation further restricts water movement. Although some wild grape species exhibit natural resistance, cultivated *V. vinifera* varieties remain highly susceptible. Upon infection, *V. vinifera* undergoes extensive transcriptional reprogramming, characterized by the activation of defense-related genes and downregulation of many metabolic pathways. These alterations highlight the complex molecular responses that enable the plant to cope up with *X. fastidiosa* infection [6].

Despite extensive research on the pathophysiological effects of PD, the dynamic transcriptomic responses of *V. vinifera* to *X. fastidiosa* remain poorly understood. Addressing this knowledge gap is crucial for elucidating the molecular mechanisms underlying susceptibility and resistance. PD progression in *V. vinifera* can be broadly divided into two phases based on symptom development and physiological changes. Phase I (early infection) marks the onset of bacterial colonization, characterized by limited xylem blockage, mild leaf scorch, and activation of defense and oxidative stress pathways. Phase II (advanced infection) involves extensive bacterial proliferation, systemic vessel occlusion, chlorosis, and dehydration, leading to severe wilting and necrosis. This transition represents a shift from active defense to metabolic decline and transcriptional suppression as the infection progresses [4,7].

Ingel et al. (2021) integrated microscopy, micro-CT, and RNA-seq to associate *X. fastidiosa* infection with tylose formation and starch depletion in *V. vinifera*, revealing broad transcriptional shifts related to ethylene signaling, cell wall biogenesis, and photosynthetic decline [6]. However, their analysis primarily describes overall expression trends without resolving the underlying regulatory network or distinguishing infection-induced changes from normal temporal variation [6].

Therefore, this study used a computational, systems-biology approach to examine transcriptional regulation and interaction networks [8], re-analyzing the GSE152164 RNA-seq dataset using a multi-contrast DESeq2 framework that compares infected and control samples across disease phases and time points, which enabled the separation of infection-specific from temporal variation [6,9]. We further constructed STRING-based protein–protein interaction (PPI) networks of differentially expressed genes (DEGs) using the Cytoscape STRING app to identify key regulatory hubs and functional modules involved in the grapevine response to *X. fastidiosa* infection.

## 2. Results

### 2.1. Identification of Differentially Expressed Genes

**Figure 1:**
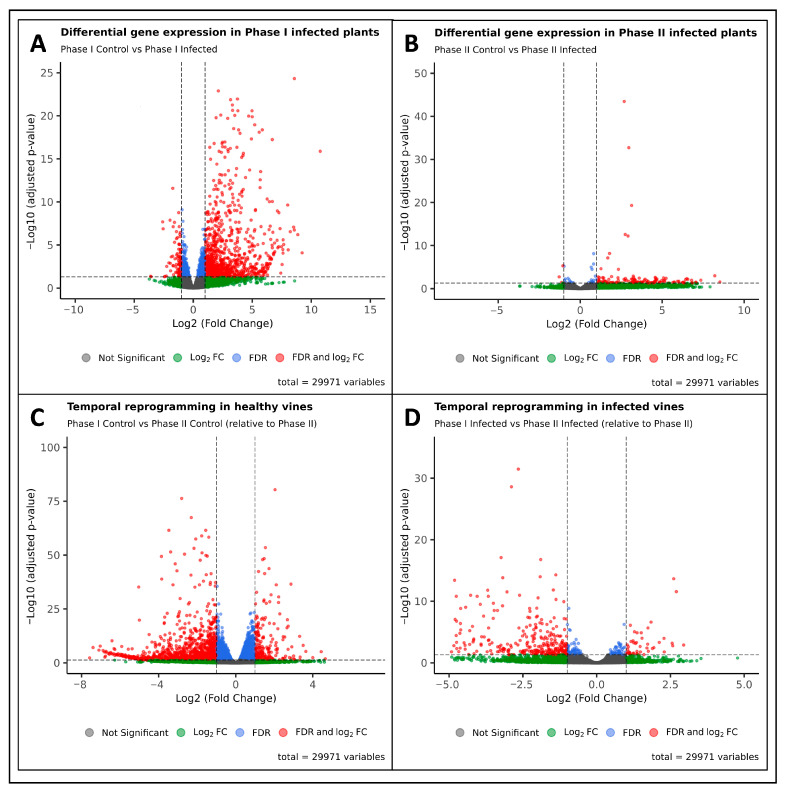
Differential gene expression analysis of *Vitis vinifera* under *Xylella fastidiosa* infection across two disease phases. (**A**) Phase I control vs. Phase I infected—differential gene expression in early infection (Phase I). (**B**) Phase II control vs. phase II Infected—differential gene expression in intermediate infection (Phase II). (**C**) Phase I control vs. Phase II control—temporal reprogramming in healthy vines. (**D**) Phase I infected vs. Phase II infected—temporal reprogramming in infected vines.

To investigate the transcriptional changes associated with *X. fastidiosa* infection in *V. vinifera*, we analyzed RNA-seq data from the Gene Expression Omnibus (GEO) dataset GSE152164, comprising 12 samples across Phases I and II under both control and infected conditions [6]. Differential expression analysis was performed using DESeq2, with the following pairwise contrasts: Phase I control vs. Phase I infected, Phase II control vs. Phase II infected, Phase I infected vs. Phase II infected, and Phase I control vs. Phase II control. Genes with a threshold of FDR < 0.05 and |log_2_ FC| ≥ 1 were considered significantly differentially expressed. Genes with log_2_ FC > 1 were classified as upregulated, and those with log_2_ FC ≤ −1 were considered as downregulated. Table 1 summarizes the DEG counts for all contrasts, and Figure 1 shows the corresponding volcano plots.

In the first pairwise comparison (Phase I control vs. Phase I onfected), 1093 DEGs were identified, comprising 986 upregulated and 107 downregulated genes, indicating a strong host response during the early phase of infection (Supplementary Material I-i). In contrast, the Phase II control vs. Phase II infected comparison yielded only 136 DEGs (133 were upregulated, and 3 were downregulated), suggesting a markedly reduced transcriptional response at the later phase of infection (Table 1) (Supplementary Material I-ii). Beyond infection-specific comparisons, we examined temporal variation within the control and infected groups. The Phase I control vs. Phase II control contrast produced the largest number of DEGs (1270), predominantly downregulated (1063) genes, reflecting developmental or age-related transcriptional shifts independent of infection (Supplementary Material I-iii). Moreover, the Phase I infected vs. Phase II infected comparison produced 336 DEGs (43 upregulated and 293 downregulated), indicating progressive reprogramming of disease-related pathways as infection advances over various stages (Table 1 and Supplementary Material I-iv).

### 2.2. Phase-Specific and Temporal Dynamics of Differentially Expressed Genes in Response to Xylella fastidiosa Infection

Table 2 provides a detailed summary of common and phase-specific DEGs identified in *V. vinifera* during *X. fastidiosa* infection. This comparative framework highlights both infection-induced and time-dependent transcriptional reprogramming, providing insights into how the grapevine defense network evolves between early and late infection stages. Among the infection–phase comparisons, the most extensive transcriptional reprogramming occurred in the “early-phase-specific response (Phase I only)” category, which encompassed 991 DEGs (884 upregulated and 107 downregulated). This strong early activation reflects rapid induction of defense-related signaling, stress-responsive transcription factors, and primary metabolic reprogramming upon pathogen perception.

The “core infection response (intersection)” category comprised 102 DEGs, all upregulated and shared between Phases I and II infected samples. These genes likely represent a sustained defense module characterized by persistent immune activation, enhanced secondary metabolism, and strengthened stress tolerance throughout infection progression.

**Figure 2:**
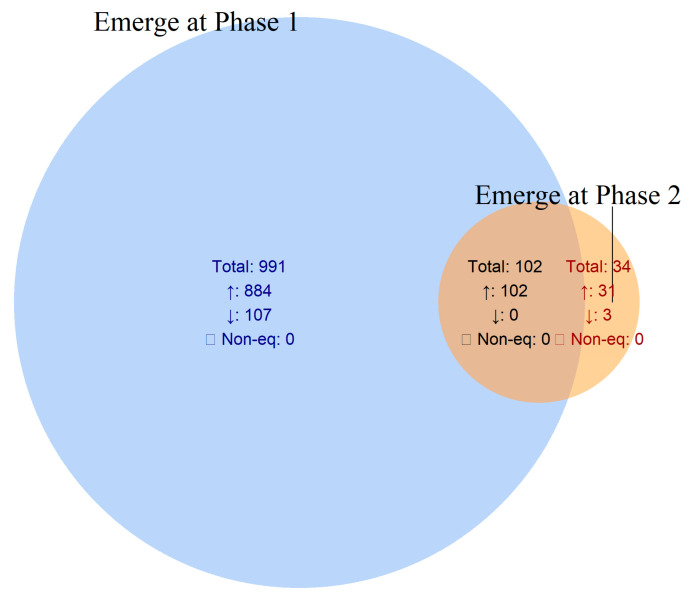
Venn diagram illustrating core and phase-specific infection DEGs in *Vitis vinifera* comparing differentially expressed genes (DEGs) between Phase I and Phase II under *Xylella fastidiosa* infection. ↑—up regulated; ↓—down regulated; ☐—Non equidirectional.

In contrast, the “late-phase-specific response (Phase II only)” subset contained only 34 DEGs (31 upregulated and 3 downregulated), suggesting that once the primary defense program is established during Phase I, Phase II involves minimal new transcriptional activation. These late-phase genes may reflect long-term defense adaptation, secondary signaling cascades, or compensatory processes that follow the initial immune response (Table 2, Figure 2).

**Figure 3:**
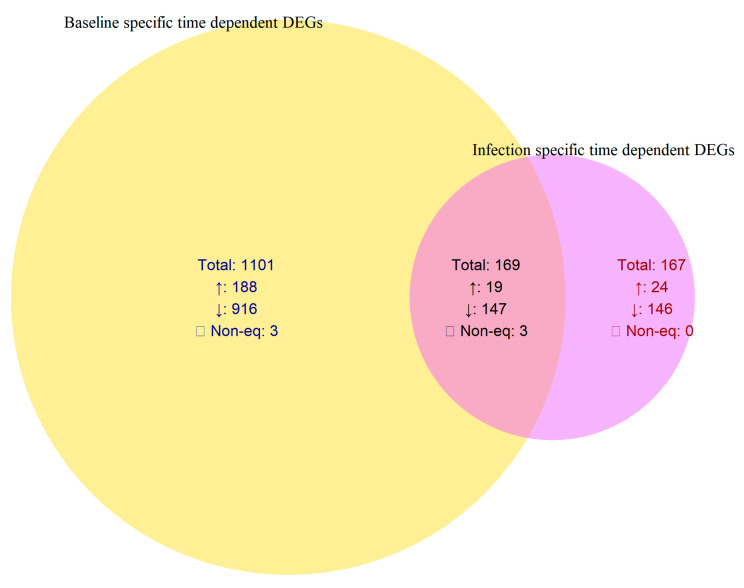
Venn diagram showing infection-specific and baseline time-dependent DEGs across phases in *Vitis vinifera* comparing differentially expressed genes (DEGs) that change over time in baseline (uninfected) and infected conditions of *Vitis vinifera* during *Xylella fastidiosa* infection. ↑—up regulated; ↓—down regulated; ☐—Non equidirectional.

In comparison, the control samples exhibited a broader set of temporally regulated genes (1101 DEGs; 188 upregulated and 916 downregulated), while infection induced a smaller but distinct subset (167 DEGs; 24 upregulated and 146 downregulated). These infection-specific temporal DEGs represent genes modulated exclusively by pathogen rather than normal developmental transitions (Table 2, Figure 3).

Overall, these findings underscore a phase-resolved transcriptional landscape in which *V. vinifera* mounts a strong early transcriptional response upon infection, maintains a subset of core responses into later phases, and undergoes a distinct temporal shift, especially widespread downregulation, unique to infected plants. This pattern reflects a tightly regulated balance between growth, energy use, and immune preparedness throughout disease progression.

### 2.3. Gene Ontology (GO) Enrichment Analysis

Gene Ontology (GO) enrichment analysis was performed using DAVID Bioinformatics Resources v6.8 [10] to identify enriched molecular functions (MFs), biological process (BPs), and cellular component (CC) terms among the DEGs (Supplementary Material I-v). GO term annotation and classification were based on the GO database, and only terms with a *p*-value < 0.05 were considered significantly enriched [11,12].

In MF enrichment, upregulated DEGs were primarily associated with ATP binding, suggesting that many DEGs are involved in energy-dependent biochemical processes. The enrichment of protein kinase activity indicates active involvement of phosphorylation-mediated signaling pathways in cellular responses, while ATPase-coupled transmembrane transporter activity highlights the role of ATP-driven transporters in maintaining ion and metabolite homeostasis across membranes. Collectively, these findings denote that energy metabolism, signal transduction, and transport-associated molecular functions are key mechanisms modulated during *X. fastidiosa* infection. Among downregulated DEGs, enriched MF terms included serine-type endopeptidase activity and transmembrane transporter activity (Table 3), reflecting reduced proteolytic and transport functions during disease progression (Supplementary Material I-v).

In BP enrichment, upregulated DEGs were predominantly involved in immune response, hormone-mediated pathways, signal transduction, defense response, lipid metabolism, and regulation of systemic acquired resistance (Table 4). No significantly enriched BP terms were detected among downregulated DEGs (Supplementary Material I-v).

For CC enrichment, upregulated DEGs were mainly localized to the plasma membrane and other membrane-associated compartments, underscoring the role of membrane-bound proteins in stress signaling and pathogen perception (Table 5). No significantly enriched CC terms were detected among downregulated DEGs (Supplementary Material I-v).

### 2.4. STRING Protein–Protein Interaction (PPI) Network and Hub-Gene Identification

To examine the functional relationships among DEGs in *V. vinifera* during *X. fastidiosa* infection, a PPI network was constructed using the STRING database [13,14] via the stringApp plugin in Cytoscape [14]. A combined score threshold of 0.40 was selected to balance interaction coverage and reliability, given the limited experimentally validated data available for *V. vinifera* in STRING. Networks generated with higher confidence thresholds (>0.70) became excessively fragmented. After construction, isolated nodes (degree = 0) and small disconnected subnetworks (<3 nodes) were removed to retain the main connected network. The resulting network exhibited sufficient density for robust topological and clustering analyses using CytoHubba (version 0.1) and MCODE (version 2.2.0).

**Figure 4:**
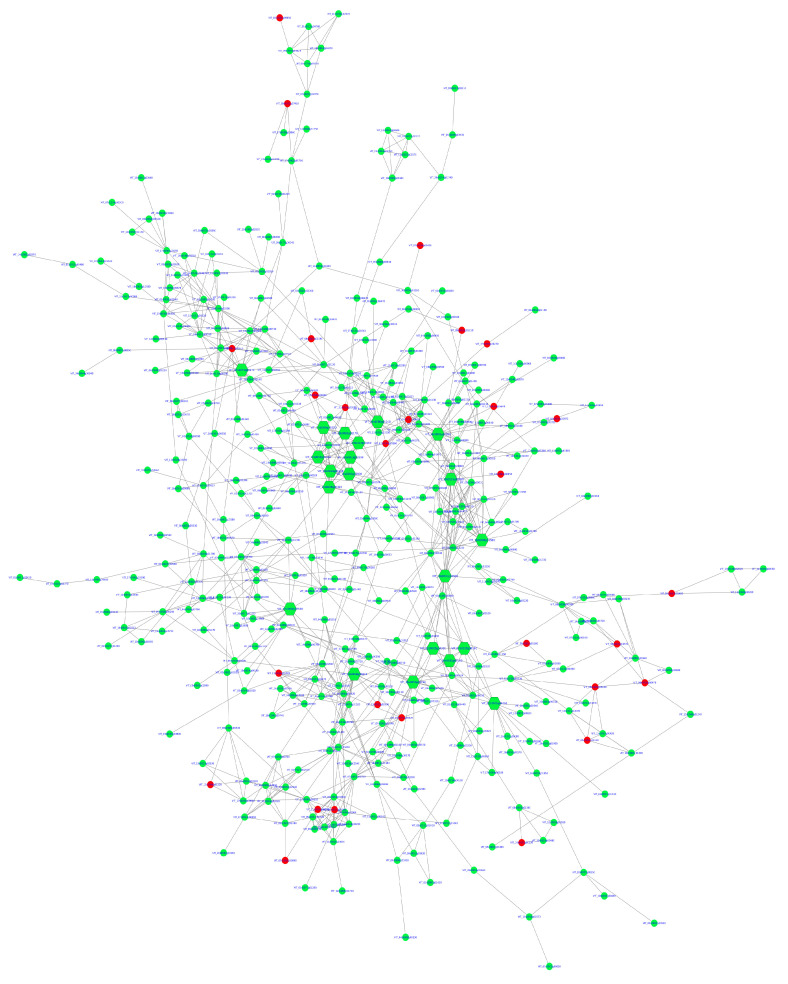
Protein–protein interaction (PPI) network of differentially expressed genes in *Vitis vinifera* during *Xylella fastidiosa* infection. Green nodes are upregulated, and red nodes are downregulated. The bigger nodes are hub genes.

The initial STRING analysis using DEGs from Phases I and II infected samples identified approximately 1126 nodes and 981 edges (Supplementary Material II-i). After removing disconnected and isolated nodes, the final core network comprised 413 nodes and 921 edges. Topological analysis revealed a clustering coefficient of 0.295, network diameter of 16, characteristic path length of 5.76, network density of 0.011, and an average of 4.46 neighbors per node (Figure 4). These parameters suggest a moderately dense network characterized by a highly interconnected central core and several loosely associated peripheral modules. The core region exhibited strong interconnectivity among genes involved in ribosomal function, defense signaling, oxidative stress response, and secondary metabolism—key biological processes activated during pathogen invasion—whereas the peripheral clusters represented stress-specific or phase-dependent responses to *X. fastidiosa* infection.

**Figure 5:**
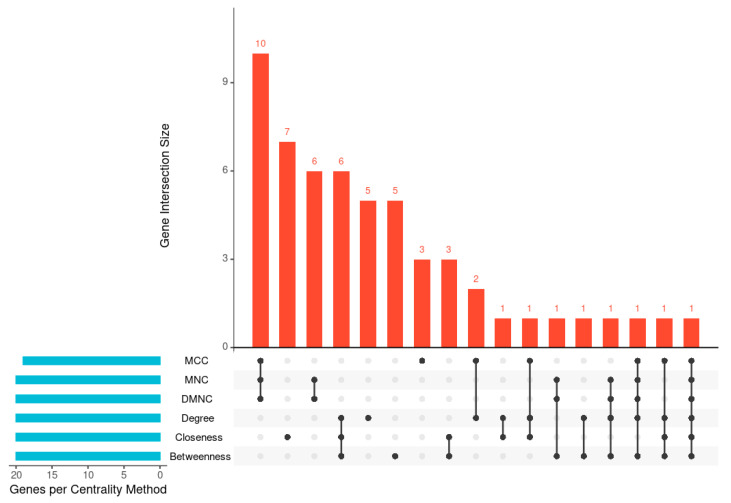
UpSet plot representing intersections among top 20 hub proteins obtained from multiple centrality-based analyses.

To identify key nodes within the PPI network (Figure 4), six CytoHubba centrality algorithms (degree, betweenness, closeness, MCC, MNC, and DMNC) were applied (Table 6). The UpSet plot (Figure 5, Supplementary Material II-ii) illustrates the overlap among the top 20 hub genes identified by these algorithms. One gene was shared across all six methods, while the largest intersection comprised ten genes common to three algorithms (MCC, MNC, and DMNC). To enhance analytical robustness, only genes considered by at least three independent algorithms were designated as high-confidence hub genes. This refinement yielded 21 consistently identified hubs representing biologically significant nodes within the *V. vinifera* defense network (Table 6 and Table 7).

The top 21 identified hub genes include VIT_16s0050g02220 (chitinase), VIT_19s0014g03660 (chlorophyll a-b binding protein, chloroplastic), VIT_18s0001g14500 (endoplasmin homolog), VIT_02s0025g04250 (thaumatin-like protein), VIT_17s0000g07560 (enhanced disease susceptibility 1), VIT_07s0031g00470 (DNA polymerase), VIT_14s0066g01240 (L-aspartate oxidase), VIT_18s0001g03910 (nitrate reductase), VIT_03s0088g00810 (SCP domain-containing protein), VIT_10s0116g01650 (adenylyl-sulphate reductase (glutathione)), VIT_16s0098g01580 (luminal-binding protein 5), VIT_02s0025g04310 (thaumatin-like protein), VIT_03s0091g00160 (basic secretory protease), VIT_07s0129g00360 (peroxidase), VIT_10s0003g02890, VIT_10s0003g02900, VIT_12s0028g00320, VIT_12s0055g01110, VIT_17s0000g06350, VIT_18s0089g01170, and VIT_19s0014g00160 (all encoding chlorophyll a-b binding protein, chloroplastic).

Among the 21 identified hub genes, three genes, viz. VIT_18s0001g14500 (endoplasmin homolog), VIT_02s0025g04250 (thaumatin-like protein), and VIT_17s0000g06350 (chlorophyll a/b-binding protein, chloroplastic) were also present among the 167 genes exhibiting infection-specific temporal expression patterns common to both phases. These hub genes are functionally associated with plant defense and environmental stress responses [15,16], highlighting their potential roles in the adaptive response of grapevine’s to *X. fastidiosa* infection.

**Figure 6:**
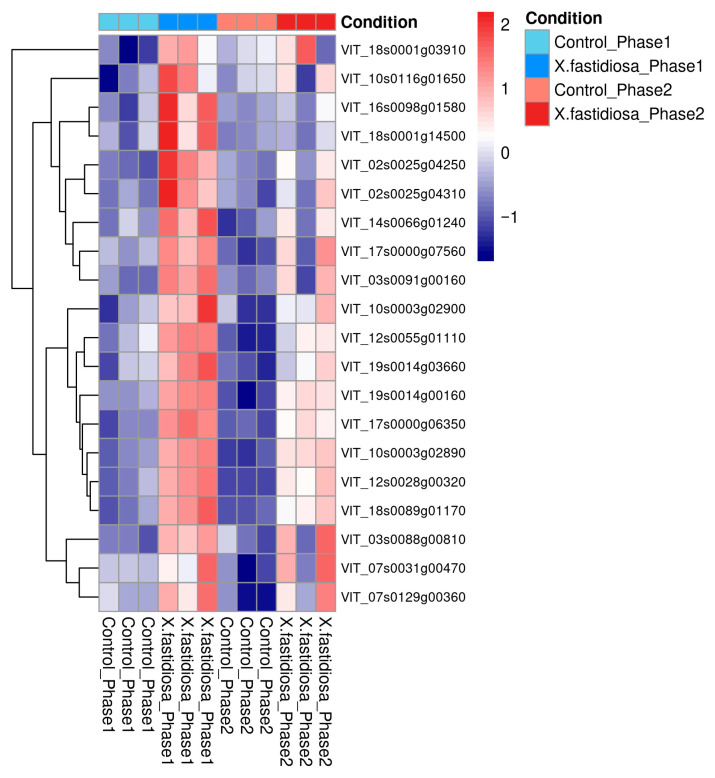
Heatmap of hub gene expression in *Vitis vinifera* under *Xylella fastidiosa* infection across phases.

A heatmap of the 21 hub genes revealed that most were upregulated in Phases I and II relative to healthy controls, indicating their sustained involvement in the infection response (Figure 6). Photosynthesis-related chlorophyll a/b binding proteins (CABs) appeared as hubs owing to network co-expression; however, they likely represent metabolic consequence nodes reflecting secondary effects of infection rather than direct defense regulators. Conversely, defense-related genes such as EDS1, thaumatin-like protein, chitinase, and endoplasmin constitute active components of the grapevine immune system, mediating signaling and stress adaptation. These functionally relevant genes represent promising molecular candidate genes and potential targets for understanding and managing PD in *V. vinifera* (Figure 6).

### 2.5. Cluster Modules Within the PPI Network

The PPI network was clustered using the MCODE algorithm with the following parameters: node score cutoff = 0.2, K-core = 2, degree cutoff = 2, and maximum depth = 100. The three most highly interconnected clusters were selected for downstream analysis (Supplementary Material I-iv) [17]. Within each cluster, *seed genes* represent highly connected nodes automatically selected by MCODE as the initial points for cluster expansion. We also examined whether these clusters contained any of the 21 previously identified hub genes, as their co-occurrence within modules would indicate significant biological importance.

Cluster 1 exhibited a cluster score of 6.000 and contained 63 nodes with a density of 22. Within this cluster, two seed genes (VIT-18s0042g00740 and VIT-14s0068g00930) and one hub gene (VIT-03s0088g00810) were identified (Supplementary Material II-iii). Cluster 2 showed the highest complexity, with a cluster score of 5.804, encompassing 148 nodes, and a density of 52. This cluster contained three seed genes (VIT-16s0050g01160, VIT-16s0098g01580, and VIT-04s0044g00860) and eight hub genes (VIT-16s0050g02220, VIT-18s001g14500, VIT-02s0025g04250, VIT-18s0001g03910, VIT-10s0115g01650, VIT-02s0025g04310, VIT-03s0091g00160, and VIT-07s0129g00360), among which one gene, VIT-16s0098g01580, functioned as a hub and seed node (Supplementary Material II-iii). Cluster 3 had a cluster score of 5.120 and comprised 64 nodes with a density of 26. This cluster contained two seed genes (VIT-01s0127g00260 and VIT-13s0019g04140) and eight hub genes (VIT-19s0014h03660, VIT-10s0003g02890, VIT-10s003g02900, VIT-12s0028g00320, VIT-12s00055g01110, VIT-17s0000g06350, VIT-18s0089g01170, and VIT-19s0014g00160) (Supplementary Material II-iii).

Functional annotation of these top-ranked clusters revealed significant enrichment in defense response, cell wall modification, and signal transduction processes, underscoring that the clustered hub genes act cooperatively to regulate multiple layers of the *V. vinifera* defense network in response to *X. fastidiosa* infection.

## 3. Discussion

Differential expression genes (DEGs) analysis remains a widely adopted approach for elucidating transcriptional changes across biological conditions, particularly when applied to high-throughput RNA sequencing data [8,18]. The study by Ingel et al. (2021) provides a foundational understanding of *Xylella fastidiosa* infection in *Vitis vinifera*, demonstrating that tylosis formation and starch depletion are associated with extensive transcriptional reprogramming across disease phases [6]. Our re-analysis advances this study by introducing a four-way contrast design that distinguishes developmental from infection-specific effects and resolves phase-dependent transcriptional dynamics. This analytical framework not only confirms the early activation of ethylene-mediated defense signaling and cell-wall remodeling but also identifies key regulatory hub genes associated with late-phase metabolic decline. Integrating PPI network topology with DEG analysis advances the dataset from a descriptive transcriptomic survey to a systems-level representation of the grapevine defense network against *X. fastidiosa*.

Using the GSE152164 dataset comprising 12 samples across Phases I and II (infected and control), DEG analysis was performed in DESeq2 [19] to evaluate four pairwise contrasts: Phase I control vs. infected, Phase II control vs. infected, Phase I control vs. Phase II control, and Phase I infected vs. Phase II infected. The results are consistent with those of Ingel et al. (2021), who report 5651 DEGs, with higher induction during the early infection stage (4636 DEGs in Phase I) and fewer in the later stage (2662 DEGs in Phase II) [6]. In our re-analysis, 1093 DEGs (986 upregulated) were detected in Phase I infected vines, indicating strong transcriptional reprogramming. In contrast, only 136 DEGs were detected in Phase II, indicating a marked decline in transcriptional activity as the disease progresses. These results remain broadly consistent with the expression patterns reported by Ingel et al. (2021) [6]. The smaller number of DEGs identified in our analysis reflects the application of stricter statistical thresholds; while Ingel et al. [6] employed an unadjusted *p* < 0.05 without a fold-change cut off, we applied a Benjamini–Hochberg adjusted *p*-value (FDR) < 0.05 and |log_2_ FC| ≥ 1, producing a more conservative and biologically reliable DEG set.

Developmental variation also exerts a strong influence in transcriptomic differences. Overall, 1270 DEGs were identified between Phases I and II controls, underscoring substantial temporal reprogramming unrelated to infection [20] (Table 2). This observation emphasizes the importance of comparing infected and control samples within each phase to minimize developmental confounding. The transition from Phase I to II in infected vines yielded 336 DEGs (43 upregulated and 293 downregulated), signifying repression of early defense genes and broad metabolic downshift (Table 2).

Phase-wise comparison of DEGs provides deeper insights into the temporal progression of *X. fastidiosa* infection in *V. vinifera*. A core set of 102 genes remained upregulated across both phases in the infected vine plants, indicating persistent defense-related activation throughout disease progression. In contrast, 991 genes were exclusively induced in Phase I, reflecting a strong but transient early response, whereas only 34 DEGs were uniquely detected in Phase II, suggesting minimal new transcriptional activation during advanced infection (Table 2; Figure 2). To distinguish infection-driven transcriptional changes from normal developmental variation, temporal DEGs in infected and control vine plants were directly compared. Among the 336 DEGs identified across the infected time course (Phase I → Phase II), 167 overlapped with DEGs from the control time course, indicating expression changes associated with normal vine maturation. The remaining 167 DEGs (24 upregulated and 146 downregulated) were unique to the infected vine plants and represent infection-specific temporal regulators. These genes likely mediate the transcriptional reprogramming underlying disease progression and symptom manifestation (Table 2; Figure 3).

Functional interpretation of these DEGs began with GO enrichment analysis, which revealed significant enrichment in defense-related molecular functions, including ATP binding, kinase activity, and transmembrane transport regulation. These results showed strong activation of signaling and energy-driven mechanisms during infection. To elucidate the broader functional context, a PPI network analysis was subsequently performed to examine how these genes integrate within grapevine defense networks.

The PPI network analysis further reinforced the centrality of early defense mechanisms in *V. vinifera* during *X. fastidiosa* infection. The STRING-based network, constructed using the StringApp in Cytoscape, exhibited a moderately dense topology with a highly connected core, indicating coordinated regulation among defense-associated genes. Application of six CytoHubba centrality algorithms (degree, betweenness, closeness, MCC, MNC, and DMNC) identified 21 high-confidence hub genes, including VIT_16s0050g02220 (chitinase), VIT_19s0014g03660 (chlorophyll a-b binding protein), VIT_18s0001g14500 (endoplasmin homolog), and VIT_02s0025g04250 (thaumatin-like protein). Furthermore, 3 of the 21 hub genes, VIT_18s0001g14500, VIT_02s0025g04250, and VIT_17s0000g06350, also appeared as infection-specific temporal DEGs, each previously implicated in defense responses [20].

Some of the identified hub genes function as key regulators of plant immunity and stress adaptation. Chitinase (VIT_16s0050g02220), a classical PR-3 protein, mediates antifungal defense and signaling and has previously been associated with grapevine responses to *X. fastidiosa* [21,22,23]. VIT_ 02s0025g04250 (thaumatin-like protein; PR-5 family) contributes to pathogen restriction via antifungal activity and cell wall modification [20,24]. VIT_17s0000g07560 (EDS1) functions as a pivotal node in salicylic acid (SA)-mediated and effector-triggered immunity [25,26]. VIT_18s0001g14500 (endoplasmin), an ER-localized HSP90 chaperone, regulates the unfolded protein response (UPR), relieving ER stress and ensuring proper folding and secretion of defense-related glycoproteins [27,28]. Additional hub genes, including VIT_18s0001g03910 (nitrate reductase) and VIT_10s0116g01650 (adenylyl-sulfate reductase), participate in nitrogen and sulfur metabolism—pathways often reprogrammed under oxidative or pathogen-induced stress [29,30]. VIT_08s0040g02590 (glutathione S-transferase) further indicates the activation of antioxidant and detoxification mechanisms that maintain cellular redox balance during infection [31].

A subset of chlorophyll a/b binding (CAB) protein isoforms, including (*VIT_10s0003g02890*, *VIT_12s0028g00320*, and related genes) also emerged among the identified hub genes. While these genes are generally downregulated during infection [32], their prominence within the interaction network likely reflects co-expression with stress-responsive modules rather than a direct involvement in defense signaling. The CAB genes displayed a biphasic expression pattern—transient induction in Phase I followed by repression in Phase II—suggesting an early photoprotective or compensatory response that diminishes as photosynthetic inhibition becomes established [15,29,33]. Thus, while CABs may act as responsive metabolic indicators, defense-related genes such as chitinase, thaumatin-like protein, and EDS1 constitute genuine functional regulators of grapevine immunity [26,34].

Overall, our systems-biology-based re-analysis of RNA-seq data reveals that *Vitis vinifera* mounts a coordinated, multi-layered defense against *Xylella fastidiosa*. This defense involves early activation of pathogenesis-related proteins, molecular chaperones, and antioxidant enzymes, followed by a progressive suppression of photosynthetic and primary metabolic processes in the late phase of infection. These results underscore the close interdependence between defense signaling and metabolic reprogramming, defining a transcriptional signature that characterizes PD progression in grapevine.

### Limitations

Although this re-analysis provides valuable systems-level insight into the transcriptional response of *Vitis vinifera*’s to *Xylella fastidiosa*, certain limitations should be acknowledged. The study lacks an independent validation dataset and experimental confirmation to substantiate the identified DEGs and hub genes. Consequently, our findings are based solely on computational inference from the RNA-seq data. In addition, the dataset includes only three biological replicates per condition (Phase I control, Phase I infected, Phase II control, and Phase II infected), which limits statistical power and reduces sensitivity for detecting subtle transcriptional changes. However, the original study by Ingel et al. (2021) [6] provides complementary experimental evidence through microscopy and physiological assays, which validates the disease-related changes in xylem structure and starch metabolism. Future studies incorporating quantitative biological replication and qualitative molecular validation of functional assays will be essential to strengthen and extend these findings.

## 4. Materials and Methods

### 4.1. Dataset Description

The RNA-seq dataset GSE152164 was retrieved from the NCBI Gene Expression Omnibus (GEO) database [6,9]. It includes transcriptomic profiles of *V. vinifera* (grapevine) stem tissues infected with *X. fastidiosa* and phosphate-buffered saline (PBS)-treated controls. Samples were collected at two clinical stages of Pierce’s disease (PD): Phase I (early infection) and Phase II (intermediate/advanced infection). Each phase represents distinct physiological conditions associated with tylose formation, starch depletion, and xylem vessel dysfunction. The dataset provides RNA-seq-derived mRNA expression counts for analyzing transcriptional changes linked to disease progression in *V*. *vinifera* xylem tissues [6,9].

### 4.2. Data Processing and Differential Expression Analysis

Differential expression analysis was performed using the DESeq2 package (v1.48.2) in R/Bioconductor (v3.22) [19]. The processed count matrix file “GSE152164_grape_raw_counts.txt.gz” from the GEO repository was used as input. Raw counts were initially normalized by DESeq2 to account for differences in sequencing depth across samples. Pairwise comparisons were conducted between the following contrasts: Phase I control vs. Phase I infected, Phase II control vs. Phase II infected, Phase I control vs. Phase II control, Phase I infected vs. Phase II infected. The DESeq2 model estimated size factors and gene-wise dispersion parameters to enable accurate normalization and differential expression inference. Multiple testing correction was performed using the Benjamini–Hochberg method, and genes with an adjusted *p*-value (FDR) < 0.05 and |log_2_ fold change| ≥ 1 were considered significantly differentially expressed [8,35]. The resulting differentially expressed genes (DEGs) were used for downstream functional enrichment and network analyses.

### 4.3. Gene Ontology and Pathway Enrichment Analysis

Gene Ontology (GO) and pathway enrichment analyses were performed using the DAVID Bioinformatics Resource (v2021, Dec 2021 release; Knowledgebase v2023q4) [10]. Enrichment was assessed across the biological process (BP), molecular function (MF), and cellular component (CC) categories, with terms showing a *p*-value < 0.05 being considered significantly enriched [11].

### 4.4. Protein–Protein Interaction (PPI) Network Construction and Analysis

To contextualize DEGs at the systems level, PPI networks were constructed using the STRING app [36] in Cytoscape (v3.10.3) [37]. Interactions with a combined confidence score ≥ 0.40 were retained. Hub genes were identified using the CytoHubba plugin, applying six centrality algorithms: betweenness, degree, closeness, MNC, MCC, and DMNC [38,39]. The top-ranked genes from each metric were compared, and those appearing in at least three algorithms were designated as high-confidence hub genes for biological interpretation [8]. The main PPI network was further clustered using the MCODE plugin (parameters: degree cutoff = 2, node score cutoff = 0.2, K-core = 2, max depth = 100) [17].

## 5. Conclusions

This study provides a comprehensive systems-level perspective on the molecular response of *V. vinifera* to *X. fastidiosa* infection, emphasizing the dynamic and phase-dependent nature of its defense regulation. Transcriptomic profiling reveals a strong and coordinated early-phase activation of defense-associated pathways involved in immune signaling, oxidative stress tolerance, and pathogen recognition, reflecting the rapid attempt of the grapevine to restrict pathogen proliferation before symptom onset. As infection progresses, the marked reduction in transcriptional activity observed in the late phase likely reflects both active suppression of host gene expression and functional deterioration of xylem tissues resulting from bacterial colonization and vascular occlusion. This temporal transition indicates that *V. vinifera* progressively redirects metabolic resources from growth to sustained defense maintenance under continuous pathogen pressure, an adaptive but metabolically expensive trade-off that manifests as hallmark symptoms of Pierce’s disease, including chlorosis, vascular blockage, and physiological decline. These identified hub genes likely function as key regulatory nodes coordinating immune activation and stress adaptation in grapevine during *X. fastidiosa* infection.

Overall, this study delineates a temporally structured defense framework in *V. vinifera* characterized by an early surge of transcriptional activation, limited late-phase induction, and a stable core of persistently upregulated defense genes. The identification of 167 infection-specific temporal DEGs highlights promising molecular candidates for early disease detection, functional validation, and genetic improvement strategies aimed at enhancing PD resistance in grapevine. Future multi-omics investigations and cultivar-level analyses are crucial to translate these molecular insights into practical tools for disease management and grapevine breeding.

## Figures and Tables

**Table 1:** Summary of differentially expressed genes (DEGs) between the phases of Pierce’s disease (PD) in grapevine (*Vitis vinifera*) caused by the bacterial pathogen *Xylella fastidiosa*.

S. No	Design Contrast	Total DEGs	Up Regulated	Down Regulated
1	Phase I control vs. Phase I infected	1093	986	107
2	Phase II control vs. Phase II infected	136	133	3
3	Phase I control vs. Phase II control	1270	207	1063
4	Phase I infected vs. Phase II infected	336	43	293

Differentially expressed genes (DEGs) were identified using thresholds of FDR < 0.05 and |log_2_ FC| ≥ 1. Genes with log_2_ FC ≥ 1 were considered upregulated; genes with log_2_ FC ≤ −1 were considered downregulated.

**Table 2:** Phase-specific and infection-specific classification of DEGs in *Vitis vinifera* during *Xylella fastidiosa* infection.

S. No	Category	Description	Up Regulated DEGs	Down Regulated DEGs	Total DEGs
1	Core infection response (intersection)	Genes differentially expressed in both Phase I and Phase II infection contrasts	102	0	102
2	Early-phase-specific response (Phase I only)	DEGs present in Phase I infected vines but absent in Phase II infected vines	884	107	991
3	Late-phase-specific response (Phase II only)	DEGs present in Phase II infected vines but absent in Phase I infected vines	31	3	34
4	Infection-specific temporal reprogramming (time-filtered)	DEGs showing temporal change in infected vines after excluding genes changing naturally in control plants	24	146	167

Upregulated DEG with |log_2_ FC| ≥ 1; Downregulated DEG with log_2_ FC ≤ −1.

**Table 3:** GO molecular function enrichment of DEGs in *Vitis vinifera* under *Xylella fastidiosa* infection.

GO ID	Molecular Function Terms	Number of Genes from DEG List	Number of Genes in Annotation
**Upregulated genes**			
GO:0005524	ATP binding	70	3499
GO:0004674	Protein serine/threonine kinase activity	27	1029
GO:0038023	Signaling receptor activity	8	126
GO:0004672	Protein kinase activity	23	1270
GO:0030246	Carbohydrate binding	9	270
GO:0004694	Rho-dependent protein serine/threonine kinase activity	7	207
GO:0004676	3-Phosphoinositide-dependent protein kinase activity	7	207
GO:0004677	DNA-dependent protein kinase activity	7	207
GO:0004711	Ribosomal protein S6 kinase activity	7	207
**Downregulated genes**			
GO:0004252	Serine-type endopeptidase activity	3	204
GO:0022857	Transmembrane transporter activity	3	376

**Table 4:** GO biological process enrichment of DEGs in *Vitis vinifera* under *Xylella fastidiosa* infection.

GO ID	Biological Process Terms	Number of Genes from DEG List	Number of Genes in Annotation
**Upregulated genes**			
GO:0048544	Recognition of pollen	13	170
GO:0006955	Immune response	10	122
GO:0009755	Hormone-mediated signaling pathway	6	73
GO:0007165	Signal transduction	11	570
GO:0006952	Defense response	12	724
GO:0006629	Lipid metabolic process	5	200
GO:0010112	Regulation of systemic acquired resistance	2	7
**Downregulated genes**			
GO:0006355	Regulation of DNA-templated transcription	4	1186
GO:0055085	Transmembrane transport	3	559
GO:0006508	Proteolysis	3	650

**Table 5:** GO cellular component enrichment of DEGs in *Vitis vinifera* under *Xylella fastidiosa* infection.

GO ID	Cellular Component Terms	Number of Genes from DEG List	Number of Genes in Annotation
**Upregulated genes**			
GO:0005886	Plasma membrane	44	2054
GO:0016020	Membrane	62	6534

**Table 6:** Top 20 hub proteins identified by different topological algorithms and centralities utilizing the CytoHubba plugin in the STRING PPI network.

	Centralities		Centralities Topological Algorithms			
S. No	Betweenness	Closeness	Degree	DMNC	MCC	MNC
1	VIT_17s0000g07560	VIT_17s0000g07560	VIT_16s0039g02040	VIT_02s0025g01610	VIT_18s0089g01170	VIT_02s0025g01610
2	VIT_04s0069g00970	VIT_08s0007g00130	VIT_17s0000g07560	VIT_02s0025g04250	VIT_07s0129g00360	VIT_02s0025g04250
3	VIT_07s0031g00470	VIT_07s0031g00470	VIT_14s0066g01060	VIT_02s0025g04310	VIT_12s0028g00320	VIT_02s0025g04310
4	VIT_08s0040g02590	VIT_07s0005g01220	VIT_07s0005g00720	VIT_02s0236g00030	VIT_02s0025g04310	VIT_02s0236g00030
5	VIT_14s0066g01240	VIT_06s0004g04470	VIT_07s0031g00470	VIT_03s0091g00160	VIT_00s0283g00030	VIT_03s0091g00160
6	VIT_02s0025g00280	VIT_00s0283g00030	VIT_18s0122g00550	VIT_07s0129g00360	VIT_10s0003g02890	VIT_07s0129g00360
7	VIT_07s0031g02390	VIT_14s0066g01240	VIT_08s0040g02590	VIT_10s0003g02890	VIT_17s0000g06350	VIT_10s0003g02890
8	VIT_18s0001g03910	VIT_02s0025g00280	VIT_05s0094g01300	VIT_10s0003g02900	VIT_03s0091g00160	VIT_10s0003g02900
9	VIT_00s0179g00340	VIT_07s0031g02390	VIT_14s0066g01240	VIT_11s0016g01020	VIT_03s0038g03410	VIT_11s0016g01020
10	VIT_13s0064g00560	VIT_13s0019g01430	VIT_18s0001g03910	VIT_12s0028g00320	VIT_05s0094g00220	VIT_12s0028g00320
11	VIT_16s0050g02220	VIT_18s0001g03910	VIT_04s0044g01100	VIT_12s0055g01110	VIT_10s0003g02900	VIT_12s0055g01110
12	VIT_18s0001g14500	VIT_16s0050g02220	VIT_16s0050g02220	VIT_13s0064g00560	VIT_04s0044g01100	VIT_13s0064g00560
13	VIT_02s0236g00130	VIT_18s0001g14500	VIT_18s0001g14500	VIT_14s0083g01100	VIT_16s0050g02220	VIT_14s0083g01100
14	VIT_04s0023g00830	VIT_04s0023g00830	VIT_13s0019g04140	VIT_15s0046g00440	VIT_18s0001g14500	VIT_15s0046g00440
15	VIT_17s0000g09790	VIT_06s0004g02620	VIT_02s0025g04250	VIT_16s0050g02220	VIT_12s0055g01110	VIT_16s0050g02220
16	VIT_02s0025g04250	VIT_11s0016g01520	VIT_19s0014g03660	VIT_17s0000g06350	VIT_13s0019g04140	VIT_17s0000g06350
17	VIT_19s0014g03660	VIT_03s0088g00810	VIT_06s0004g02620	VIT_18s0089g01170	VIT_19s0014g00160	VIT_18s0089g01170
18	VIT_16s0022g00060	VIT_04s0044g00860	VIT_03s0088g00810	VIT_19s0014g00160	VIT_19s0014g03660	VIT_19s0014g00160
19	VIT_03s0088g00810	VIT_16s0098g01580	VIT_16s0098g01580	VIT_19s0014g01640	VIT_00s2171g00010	VIT_19s0014g01640
20	VIT_10s0116g01650	VIT_10s0116g01650	VIT_10s0116g01650	VIT_19s0014g03660	VIT_16s0098g01580	VIT_19s0014g03660

The color-highlighted genes are present in more than three columns, as indicated by color code: blue color denotes presence in six columns, gray color denotes presence in five columns, black color denotes presence in four columns, and red color denotes presence in four columns.

**Table 7:** List of hub genes identified from STRING PPI work.

S. No	Gene Symbol	Gene Name
1	VIT_16s0050g02220	Chitinase
2	VIT_19s0014g03660	Chlorophyll a-b binding protein
3	VIT_18s0001g14500	Endoplasmin homolog
4	VIT_02s0025g04250	Thaumatin-like protein
5	VIT_17s0000g07560	Enhanced disease susceptibility 1
6	VIT_07s0031g00470	DNA polymerase
7	VIT_14s0066g01240	L-aspartate oxidase
8	VIT_18s0001g03910	Nitrate reductase
9	VIT_03s0088g00810	SCP domain-containing protein
10	VIT_10s0116g01650	Adenylyl-sulphate reductase [glutathione]
11	VIT_16s0098g01580	Luminal-binding protein 5
12	VIT_02s0025g04310	Thaumatin-like protein
13	VIT_03s0091g00160	Basic secretory protease
14	VIT_07s0129g00360	Peroxidase
15	VIT_10s0003g02890	Chlorophyll a-b binding proteins
16	VIT_10s0003g02900	Chlorophyll a-b binding proteins
17	VIT_12s0028g00320	Chlorophyll a-b binding proteins
18	VIT_12s0055g01110	Chlorophyll a-b binding proteins
19	VIT_17s0000g06350	Chlorophyll a-b binding proteins
20	VIT_18s0089g01170	Chlorophyll a-b binding proteins
21	VIT_19s0014g00160	Chlorophyll a-b binding proteins

## Data Availability

The original contributions presented in this study are included in the article and Supplementary Materials. Further inquiries can be directed to the corresponding author.

## Supplementary Materials

The following supporting information can be downloaded at: https://www.mdpi.com/article/10.3390/ijms262211040/s1.

## Abbreviations

PD	Pierce’s disease
PPI	Protein–protein network
CC	Cellular component
BP	Biological process
MF	Molecular function
DEG	Differentially expressed gene
GO	Gene Ontology
GEO	Gene Expression Omnibus
CAB	Chlorophyll a/b binding

## References

[1] Rapicavoli J., Ingel B., Blanco-Ulate B., Cantu D., Roper C. (2018). Xylella Fastidiosa: An Examination of a Re-Emerging Plant Pathogen. Mol. Plant Pathol..

[2] Kyrkou I., Pusa T., Ellegaard-Jensen L., Sagot M.-F., Hansen L.H. (2018). Pierce’s Disease of Grapevines: A Review of Control Strategies and an Outline of an Epidemiological Model. Front. Microbiol..

[3] Hopkins D.L., Purcell A.H. (2002). Xylella Fastidiosa: Cause of Pierce’s Disease of Grapevine and Other Emergent Diseases. Plant Dis..

[4] Deyett E., Pouzoulet J., Yang J.-I., Ashworth V.E., Castro C., Roper M.C., Rolshausen P.E. (2019). Assessment of Pierce’s Disease Susceptibility in *Vitis vinifera* Cultivars with Different Pedigrees. Plant Pathol..

[5] Giménez-Romero A., Galván J., Montesinos M., Bauzà J., Godefroid M., Fereres A., Ramasco J.J., Matías M.A., Moralejo E. (2022). Global Predictions for the Risk of Establishment of Pierce’s Disease of Grapevines. Commun. Biol..

[6] Ingel B., Reyes C., Massonnet M., Boudreau B., Sun Y., Sun Q., McElrone A.J., Cantu D., Roper M.C. (2021). Xylella Fastidiosa Causes Transcriptional Shifts That Precede Tylose Formation and Starch Depletion in Xylem. Mol. Plant Pathol..

[7] Choi H.-K., Iandolino A., da Silva F.G., Cook D.R. (2013). Water Deficit Modulates the Response of *Vitis vinifera* to the Pierce’s Disease Pathogen Xylella Fastidiosa. Mol. Plant-Microbe Interact. MPMI.

[8] Adaikalasamy P., Kumari S., Elizabeth Jacob S., Bhuiyan S., Baskaran R.R., Sampath S., Devanesan S. (2024). Integrative Bioinformatics Analysis of Transcriptomic Data from CD8+ T Cells in Systemic Lupus Erythematosus. J. King Saud Univ.—Sci..

[9] Clough E., Barrett T., Wilhite S.E., Ledoux P., Evangelista C., Kim I.F., Tomashevsky M., Marshall K.A., Phillippy K.H., Sherman P.M. (2024). NCBI GEO: Archive for Gene Expression and Epigenomics Data Sets: 23-Year Update. Nucleic Acids Res..

[10] Dennis G., Sherman B.T., Hosack D.A., Yang J., Gao W., Lane H.C., Lempicki R.A. (2003). DAVID: Database for Annotation, Visualization, and Integrated Discovery. Genome Biol..

[11] Huang D.W., Sherman B.T., Lempicki R.A. (2009). Systematic and Integrative Analysis of Large Gene Lists Using DAVID Bioinformatics Resources. Nat. Protoc..

[12] Ashburner M., Ball C.A., Blake J.A., Botstein D., Butler H., Cherry J.M., Davis A.P., Dolinski K., Dwight S.S., Eppig J.T. (2000). Gene Ontology: Tool for the Unification of Biology. Nat. Genet..

[13] Szklarczyk D., Nastou K., Koutrouli M., Kirsch R., Mehryary F., Hachilif R., Hu D., Peluso M.E., Huang Q., Fang T. (2025). The STRING Database in 2025: Protein Networks with Directionality of Regulation. Nucleic Acids Res..

[14] Doncheva N.T., Morris J.H., Gorodkin J., Jensen L.J. (2019). Cytoscape StringApp: Network Analysis and Visualization of Proteomics Data. J. Proteome Res..

[15] Wei Y., Lu X., Bao J., Zhang C., Yan H., Li K., Gong M., Li S., Ma S. (2022). Identification and Expression Analysis of Chlorophyll a/b Binding Protein Gene Family in Grape (*Vitis vinifera*). Physiol. Mol. Biol. Plants Int. J. Funct. Plant Biol..

[16] Lu X., Ma L., Zhang C., Yan H., Bao J., Gong M., Wang W., Li S., Ma S., Chen B. (2022). Grapevine (*Vitis vinifera*) Responses to Salt Stress and Alkali Stress: Transcriptional and Metabolic Profiling. BMC Plant Biol..

[17] Bader G.D., Hogue C.W. (2003). An Automated Method for Finding Molecular Complexes in Large Protein Interaction Networks. BMC Bioinform..

[18] Bobrovskikh A.V., Zubairova U.S., Doroshkov A.V. (2025). Identification of Key Differentially Expressed Genes in Arabidopsis Thaliana Under Short- and Long-Term High Light Stress. Int. J. Mol. Sci..

[19] Love M.I., Huber W., Anders S. (2014). Moderated Estimation of Fold Change and Dispersion for RNA-Seq Data with DESeq2. Genome Biol..

[20] van Loon L.C., Rep M., Pieterse C.M.J. (2006). Significance of Inducible Defense-Related Proteins in Infected Plants. Annu. Rev. Phytopathol..

[21] Schlumbaum A., Mauch F., Vögeli U., Boller T. (1986). Plant Chitinases Are Potent Inhibitors of Fungal Growth. Nature.

[22] Colas S., Afoufa-Bastien D., Jacquens L., Clément C., Baillieul F., Mazeyrat-Gourbeyre F., Monti-Dedieu L. (2012). Expression and In Situ Localization of Two Major PR Proteins of Grapevine Berries during Development and after UV-C Exposition. PLoS ONE.

[23] Chakraborty S., Nascimento R., Zaini P.A., Gouran H., Rao B.J., Goulart L.R., Dandekar A.M. (2016). Sequence/Structural Analysis of Xylem Proteome Emphasizes Pathogenesis-Related Proteins, Chitinases and β-1, 3-Glucanases as Key Players in Grapevine Defense against Xylella Fastidiosa. PeerJ.

[24] Misra R.C., Sandeep, Kamthan M., Kumar S., Ghosh S. (2016). A Thaumatin-like Protein of Ocimum Basilicum Confers Tolerance to Fungal Pathogen and Abiotic Stress in Transgenic Arabidopsis. Sci. Rep..

[25] Feng Y., Tang M., Xiang J., Liu P., Wang Y., Chen W., Fang Z., Wang W. (2023). Genome-Wide Characterization of L-Aspartate Oxidase Genes in Wheat and Their Potential Roles in the Responses to Wheat Disease and Abiotic Stresses. Front. Plant Sci..

[26] Gao F., Dai R., Pike S.M., Qiu W., Gassmann W. (2014). Functions of EDS1-like and PAD4 Genes in Grapevine Defenses against Powdery Mildew. Plant Mol. Biol..

[27] Liu J.-X., Howell S.H. (2016). Managing the Protein Folding Demands in the Endoplasmic Reticulum of Plants. N. Phytol..

[28] Howell S.H. (2013). Endoplasmic Reticulum Stress Responses in Plants. Annu. Rev. Plant Biol..

[29] Purcino R.P., Medina C.L., Martins de Souza D., Winck F.V., Machado E.C., Novello J.C., Machado M.A., Mazzafera P. (2007). Xylella Fastidiosa Disturbs Nitrogen Metabolism and Causes a Stress Response in Sweet Orange Citrus Sinensis Cv. Pera. J. Exp. Bot..

[30] Fu Y., Tang J., Yao G.-F., Huang Z.-Q., Li Y.-H., Han Z., Chen X.-Y., Hu L.-Y., Hu K.-D., Zhang H. (2018). Central Role of Adenosine 5′-Phosphosulfate Reductase in the Control of Plant Hydrogen Sulfide Metabolism. Front. Plant Sci..

[31] Dixon D.P., Lapthorn A., Edwards R. (2002). Plant Glutathione Transferases. Genome Biol..

[32] Han X., Han S., Li Y., Li K., Yang L., Ma D., Fang Z., Yin J., Zhu Y., Gong S. (2023). Double Roles of Light-Harvesting Chlorophyll *a*/*b* Binding Protein TaLhc2 in Wheat Stress Tolerance and Photosynthesis. Int. J. Biol. Macromol..

[33] Zaini P.A., De La Fuente L., Hoch H.C., Burr T.J. (2009). Grapevine Xylem Sap Enhances Biofilm Development by Xylella Fastidiosa. FEMS Microbiol. Lett..

[34] Ali M., Li Q.-H., Zou T., Wei A.-M., Gombojab G., Lu G., Gong Z.-H. (2020). Chitinase Gene Positively Regulates Hypersensitive and Defense Responses of Pepper to Colletotrichum Acutatum Infection. Int. J. Mol. Sci..

[35] Benjamini Y., Hochberg Y. (1995). Controlling the False Discovery Rate: A Practical and Powerful Approach to Multiple Testing. J. R. Stat. Soc. Ser. B Methodol..

[36] Franceschini A., Szklarczyk D., Frankild S., Kuhn M., Simonovic M., Roth A., Lin J., Minguez P., Bork P., von Mering C. (2013). STRING v9.1: Protein-Protein Interaction Networks, with Increased Coverage and Integration. Nucleic Acids Res..

[37] Shannon P., Markiel A., Ozier O., Baliga N.S., Wang J.T., Ramage D., Amin N., Schwikowski B., Ideker T. (2003). Cytoscape: A Software Environment for Integrated Models of Biomolecular Interaction Networks. Genome Res..

[38] Chin C.-H., Chen S.-H., Wu H.-H., Ho C.-W., Ko M.-T., Lin C.-Y. (2014). cytoHubba: Identifying Hub Objects and Sub-Networks from Complex Interactome. BMC Syst. Biol..

[39] Ahmed W., Wang Y., Ji W., Liu S., Zhou S., Pan J., Li Z., Wang F., Wang X. (2025). Unraveling the Mechanism of the Endophytic Bacterial Strain Pseudomonas Oryzihabitans GDW1 in Enhancing Tomato Plant Growth Through Modulation of the Host Transcriptome and Bacteriome. Int. J. Mol. Sci..

